# Routine surveillance endomyocardial biopsy for the detection of cellular rejection beyond 2 years after cardiac transplantation

**DOI:** 10.1186/1749-8090-10-S1-A288

**Published:** 2015-12-16

**Authors:** Christian Heim, Fatos Ballazhi, Thorsten Zielezinski, Markus Kondruweit, Michael Weyand, Rene Tandler

**Affiliations:** 1 Department of Cardiac Surgery, Friedrich-Alexander University, Erlangen, 91054, Germany

## Background/Introduction

Endomyocardial biopsy (EMB) is widely used for routine surveillance of cardiac allograft rejection. The need for continued EMB beyond the first year after cardiac transplantation is controversial.

## Aims/Objectives

The aim of this study was to investigate the use of EMB in monitoring long term surviving heart transplant recipients.

## Method

We conducted a retrospective chart review of all patients at our center 2 years or more after heart transplantation. 154 HTx patients between 2000-2012 were included in this study. Significant cellular rejection was defined as grade 2R or 3R using ISHLT nomenclature. Patients were analyzed assessing immunosuppressive regimen and procedural related complications.

## Results

Of 154 cardiac transplant patients, 110 (71.4%) had a follow-up of more than 2 years. Interestingly, 17 of these long-term survivors of cardiac transplantation developed at least 1 episode of significant late (>2 years after Tx) cellular rejection (15.5%). Analyzing the respective immunosuppressive regimen showed increased number of calcineurin inhibitor (CNI)-free regimen (64.7%) in patients rejecting late after heart transplantation. The overall incidence of procedural related complications was low (1.0%) and none was life-threatening.

## Discussion/Conclusion

The above data demonstrates that endomyocardial biopsies continue to detect clinically significant rejection beyond 2 years after cardiac transplantation. Late rejection was not depending on previous episodes of early cellular rejections. Therefore, we recommend long-term routine endomyocardial biopsies in cardiac transplant recipients especially in patients at high risk due to the immunosuppressive regimen.

